# Guidelines on uncomplicated urinary tract infections are difficult to follow: perceived barriers and suggested interventions

**DOI:** 10.1186/1471-2296-11-51

**Published:** 2010-06-28

**Authors:** Marjolein Lugtenberg, Jako S Burgers, Judith M Zegers-van Schaick, Gert P Westert

**Affiliations:** 1 Scientific Centre for Transformation in Care and Welfare (Tranzo), Tilburg University, PO Box 90153, 5000 LE Tilburg, the Netherlands; 2 Scientific Institute for Quality of Healthcare (IQ Healthcare), Radboud University Nijmegen Medical Centre, PO Box 9101, 6500 HB Nijmegen, the Netherlands; 3 Amphia hospital, Department of Cardiology, PO Box 90158, 4800 RK, Breda, the Netherlands; 4 National Institute for Public Health and the Environment (RIVM), PO Box 1, 3720 BA Bilthoven, the Netherlands

## Abstract

**Background:**

Urinary tract infections (UTI) are among the most common health problems seen in general practice. Evidence-based guidelines on UTI are available, but adherence to these guidelines varies widely among practitioners for reasons not well understood. The aim of this study was to identify the barriers to the implementation of a guideline on UTI perceived by Dutch general practitioners (GPs) and to explore interventions to overcome these barriers.

**Methods:**

A focus group study, including 13 GPs working in general practices in the Netherlands, was conducted. Key recommendations on diagnosis and treatment of uncomplicated UTI were selected from the guideline. Barriers to guideline adherence and possible interventions to address these barriers were discussed. The focus group session was audio-taped and transcribed verbatim. Barriers were classified according to an existing framework.

**Results:**

Lack of agreement with the recommendations, unavailable and inconvenient materials (i.e. dipslides), and organisational constraints were perceived as barriers for the diagnostic recommendations. Barriers to implementing the treatment recommendations were lack of applicability and organisational constraints related to the availability of drugs in pharmacies. Suggested interventions were to provide small group education to GPs and practice staff members, to improve organisation and coordination of care in out of hour services, to improve the availability of preferred dosages of drugs, and to pilot-test guidelines regionally.

**Conclusions:**

Despite sufficient knowledge of the recommendations on UTI, attitudinal and external barriers made it difficult to follow them in practice. The care concerning UTI could be optimized if these barriers are adequately addressed in implementation strategies. The feasibility and success of these strategies could be improved by involving the target group of the guideline in selecting useful interventions to address the barriers to implementation.

## Background

Urinary tract infection (UTI) is one of the most common health problems for which patients seek medical care. It is responsible for about 1% of all general practitioner (GP) consultations in the UK [1] and results in approximately 7.9 million physician visits in the United States annually [2]. In the Netherlands, UTIs rank 8^th ^on the list of most common reasons for visiting a GP and also account for 1% of all visits [3]. Most of these are uncomplicated UTIs, defined as cystitis in non-pregnant adult women. In the Dutch healthcare system, uncomplicated UTI is diagnosed and treated by GPs and rarely needs specialist care.

To optimize care concerning uncomplicated UTI, evidence-based clinical guidelines have been developed in several countries [4]. However, adherence to these guidelines has shown to be far from optimal. In a large study among a representative sample of general practices in the Netherlands, it was found that GPs followed the guideline with respect to the treatment of UTIs in 42% of the cases and that the level of adherence varied widely (0-95%) between practices [5]. A recent study showed that Dutch GPs treated UTIs according to the guideline in 50% of the cases [6]. In other countries similar levels of adherence regarding the treatment of UTIs were found [7-9]. Reasons underlying GPs suboptimal behaviour are thus far poorly understood [6-9].

An analysis of barriers to the implementation of a guideline is considered to be a first important step in improving guideline adherence [10,11]. The barriers identified can subsequently be used to develop tailored implementation strategies. Little is known on how to translate the identified barriers into tailored interventions. Several studies have shown that the choice of a specific intervention in practice is not necessarily based on the analysis of barriers [12], but is often determined by personal preferences or familiarity with some types of interventions [10,13]. Moreover, the target users of the guideline are usually not involved in selecting implementation strategies to improve adherence [14].

The aim of our study was to identify the perceived barriers to implementation of a national guideline on uncomplicated UTI among Dutch GPs and to explore interventions that could address these barriers. By conducting an in-depth analysis of barriers and possible interventions to achieving change among the target group, we aimed to provide useful suggestions for improving the care concerning uncomplicated UTI.

## Methods

### Setting: the GP in the Dutch Healthcare system

In the Netherlands, the GP has a central role in primary care as both family physician and gatekeeper to specialist care. Nearly all (99%) Dutch citizens are registered with a GP. Consultation of GPs is free and co-payments for drugs and other services are very low compared to other countries [15]. GPs also deliver primary care in out of hours services, which are organised by regional collaborative groups [16]. Almost all GPs are member of the Dutch College of General Practitioners (NHG), which is responsible for guideline development, education, and practice support [17]. Since late 1980s the NHG has developed more than 80 national guidelines for general practice, including a guideline on UTI.

### Study design

We conducted a focus group session among a sample of Dutch GPs (N = 13) working in practice. Focus groups are considered as useful methods to explore cognitions and motivations underlying behaviour, providing detailed information on perceived barriers and resistance [18-21]. In addition, focus groups often encourage creative thinking, which can be particularly useful in exploring interventions to address the barriers to guideline adherence. The focus group session on UTI was part of a larger study on guideline implementation; the results of this study were published elsewhere [22].

### Selection of participants

GPs were recruited by Stichting KOEL, a foundation responsible for continuing medical education for GPs in the South-Western part of the Netherlands [23], through advertising in their electronic newsletter and website. The GPs were offered continuing medical education accreditation points (2 hours). One week in advance to the session, they received a copy of a summary of the guideline. All thirteen GPs that registered for the UTI focus group session, participated.

### UTI guideline and key recommendations

The UTI guideline developed by the NHG was published in 1989 [24] and updated in 1999 [25] and in 2005 [26]. In 1999 the recommendations on diagnosing UTI changed in preferring the dipslide method above microscopic urinary investigation. In 2005, the classification of diagnostic categories changed, i.e. only UTIs in patients without particular risk factors or concomitant diseases, in otherwise healthy, non-pregnant women, are considered as uncomplicated. Due to increased bacterial resistance to trimethophrim, nitofurantoin is recommended as the drug of first choice and the recommended duration of treatment with nitrofurantoin was extended from 3 to 5 days. A summary of the 2005 guideline concerning uncomplicated UTI is provided in Table 1.

**Table 1:** Summary of the Dutch guideline on uncomplicated UTI 2005 (second revision)

- History taking is paramount for diagnosis of UTI. If history is typical, urinalysis is not necessary in non pregnant, otherwise healthy women.
- Urinalysis consists of a nitrite dipstick test, followed by a urine dipslide test in case of a negative nitrite test.
- A UTI is defined as a positive nitrite test or a dipslide with at least 10^4 ^colony-forming units per ml urine.
- If a woman has complaints similar to an earlier uncomplicated UTI, empirical treatment can be considered without urinalysis.
- In uncomplicated urinary tract infections, i.e. cystitis in non-pregnant, otherwise healthy women, nitrofurantoin (5 days) is the drug of first choice. In case of hypersensitivity, trimethoprim (3 days) is recommended.
- Fluorochinolonen should only be prescribed based on the specific results of a urine culture including antibiotic resistance pattern.

### Focus group session

The GPs had a semi-structured discussion about barriers to the implementation of the recommendations of the UTI guideline. They were also asked to suggest interventions to address the barriers to implementation. In this study implementation is defined as the introduction of an innovation into daily routine; this demands removal of barriers to change by using strategies that have been shown to be effective in practice [27]. We therefore considered all potential barriers that may hinder physicians from following the guideline recommendations consistently in practice. A checklist with relevant topics based on an existing framework of barriers [28], including guideline knowledge, attitude towards the guideline, external barriers to guideline adherence and suggested interventions to address the barriers, was used to structure the discussion. The session was chaired by a GP with 15 years of experience in general practice (JB), and co-chaired by one of the authors (ML). The session was held at Stiching KOEL in Zwijndrecht, the Netherlands in 2008, and was audio taped.

### Data analysis and synthesis

The audio taped discussion was transcribed verbatim. We used Cabana's framework of guideline barriers [28] to classify and analyse the data. According to this framework, guideline adherence can be affected by three main categories of barriers, which are divided into several subcategories of barriers: 1) knowledge-related barriers (lack of awareness and lack of familiarity), 2) attitude-related barriers (lack of agreement, lack of self-efficacy, lack of outcome expectancy and lack of motivation/inertia of previous practice) and 3) external barriers that limit physicians' ability to apply the guideline in practice (guideline factors, environmental factors and patient factors).

Two of the authors (ML and JZ) independently studied the transcripts and classified comments about barriers according to the framework of Cabana et al. [28]. If necessary, additional types of barriers, not covered by the existing framework, were formulated. Discrepancies in classification between the two authors were discussed until consensus was reached.

## Results

### Description of participants

Most of the participants were male (69%), were aged between 45 and 54 years (54%), were working in a group practice (46%) and had their practice located in a rural area or small town (54%). Compared to the Dutch population of GPs [29], GPs working in group practices and in practices located in a rural area or small town were slightly overrepresented.

### Perceived barriers to diagnosis of UTIs

The participants were familiar with the recommendations on diagnosis. One of the perceived barriers to diagnosing UTI was a lack of agreement with the guideline recommendation (Table 2). Some GPs disagreed with performing the nitrite dipstick test only and preferred to combine this test with leukocyte esterase dipstick test, which is often available on the same strip. Reason for disagreement was that they argued the evidence supporting this recommendation.

**Table 2:** Barriers to adherence and suggested interventions to improve adherence to recommendations on diagnosing uncomplicated UTI

	Perceived barriers	Suggested interventions
***Barriers related to knowledge***	No barriers	Not applicable
***Barriers related to attitudes***		
*Lack of agreement with recommendation*	Lack of evidence: Arguing supporting evidence for performing only the nitrite dipstick test (rather than combining it with leukocyte esterase dipstick test). Lack of applicability: Belief that benefits do not outweigh patients' discomfort due to time to wait for results of dipslide, particularly in case of serious complaints.	Small group education: Provide detailed information on supporting evidence of recommendations and discuss recommendations in peer review groups.
***External barriers***		
Environmental factors		
*Organisational constraints*	Within organisation: - Difficult to change routines of practice assistants. - Not possible to apply the dipslide on Friday (nobody available to read the results on Saturday). Outside organisation: Difficult to apply dipslide in weekend in out of hour service, particularly on Sunday (nobody available to read the results on Monday).	Dealing with diagnosing UTI in out of hours: - Develop regional protocols for weekend based on local agreements with hospitals. - Provide method for arranging local agreements in national guideline. - Adapt guideline recommendation to current practice by not recommending using dipslides in out of hour services.
* Lack of/inconvenient resources/materials*	Lack of availability/inconvenience: Dipslides are inconvenient and difficult to apply in practice and not everywhere available.	

*"I am always using the whole strip because of the evidence. Though a positive leukocyte test does not provide much information, a negative leukocyte test does. That's why I am in favour of still performing a leukocyte test"*.

The complete dipstick test is often used in practice to replace the dipslide, in particular when symptoms are mild and patients agree with watchful waiting if the test is negative. The GPs also questioned the applicability of the recommendation concerning the use of the dipslide in case of serious or severe complaints. In these cases, GPs did not always apply the dipslide.

*"It [diagnosis] also depends on the severity of the complaints. If the patient has serious complaints and she should have waited until the next day for the results, I usually do not use the dipslide method at all"*.

In addition, some GPs mentioned that dipslides are inconvenient to use in practice and not always available, both in their own practice and in out of hours services.

*"I am aware of the recommendation, but I think the dipslide is inconvenient to apply in practice and that is why I don't use it. Moreover, I could not even use it, because I do not have a dipslide in my practice"*.

They also mentioned organisational barriers to performing dipslides on Friday and during the weekends in out of hour services.

*"You can basically use the dipslide only from Monday until Thursday [....].If you have a patient on Sunday, there is no one [practice assistant or GP] available to read the results of the dipslide on Monday"*.

Another barrier within the own organisation related to the recommendation on diagnosis was that routines and habits of practice assistants need to be changed, for instance, that in the case of symptoms of recurrent uncomplicated UTIs, empirical treatment could be started without urinalysis:

*"The big change in our practice was that assistants should first take patient's history before urinalysis, and that urinalysis is not always indicated. We tried to write a new protocol for them, but the assistants argued that they often receive the urine from patients without knowing the history of the patient"*.

### Suggested interventions to improve adherence

To improve guideline adherence concerning the diagnosis of uncomplicated UTI, GPs suggested that more efforts are needed to raise awareness of the supporting evidence of guideline recommendations (Table 2). They emphasized that it is insufficient to just disseminate the guideline, and that they need to be convinced by strong arguments why they should change their routines. According to the GPs, discussing guideline recommendations and the accompanying scientific background information in small peer review groups would be a useful method.

*"In my experience, it is not enough to just read the guideline or guideline summary. Then I will not be convinced and will not change my routines [...]. You really need to 'do' something with it, such as discussing the guideline recommendations in small group of GPs, exchanging arguments, and discussing pros and cons. Then it will have an effect"*.

To reduce organisational constraints, GPs suggested that it might be useful to develop protocols specifically targeting practice assistants:

*"It would be really helpful to develop a protocol for practice assistants, in addition to the guideline. Because they do most of the work! "*.

In addition, they mentioned that it would be useful to develop regional protocols on diagnosing UTI in out of hours services.

*"There is a need for information on how to deal with diagnosing UTIs in the weekend. An option is to develop local protocols that include agreements with [specialists in] local hospitals [..]. I also think that the national guideline should pay attention to this issue{....], for instance by suggesting to develop a protocol including arrangements with hospitals"*.

Finally, it was suggested to adapt the guideline recommendation to current practice, by not recommending the use of dipslides in out of hour services.

*"My opinion is that dipslides should not be used at all in out of hour services"*.

### Perceived barriers to the treatment of uncomplicated UTIs

Barriers related to the treatment of uncomplicated UTIs were related to lack of agreement with the recommendation and to environmental factors (Table 3). GPs often prescribe trimethoprim rather than nitrofurantoin as a first choice drug because they belief that the benefits of prescribing nitrofurantoin do not outweigh the discomfort for patients:

**Table 3:** Barriers to adherence and suggested interventions to improve adherence to recommendations on treatment of uncomplicated UTI

	Perceived barriers	Suggested interventions
***Barriers related to knowledge***	No barriers	Not applicable
***Barriers related to attitudes***		
*Lack of agreement specific recommendation*	Lack of applicability: - Belief that recommendation is not applicable to patient population due to local patterns of bacterial resistance. - Belief that benefits do not outweigh patients' discomfort (taking drug 4 times a day) of prescribing drug of first choice.	Pilot-testing of guidelines on resistance: Guidelines should be tested on regional patterns of bacterial resistance of the recommended drugs. Availability of user friendly dosage of drugs: The recommended drugs should be available in a user friendly dosage.
***External barriers***		
Environmental factors		
*Organisational constraints*	Outside organisation: Recommended drugs are not available in the preferred dosage (nitrofurantoin).	Idem

"Nitrofurantoin needs to be taken four times a day. And I think it makes a big difference just taking one tablet in the evening or taking four tablets a day"

Some GPs disagreed with using trimethoprim as second choice drug due to lack of applicability to their practice population. They mentioned that they could not prescribe trimethoprim (in case of hypersensitivity for nitrofurantoin) because of regional patterns of resistance:

"We changed our [drug prescription] policy due to bacterial resistance. Trimethoprim is third choice now. I even know a city where it is just not an option anymore!"

GPs also reported organisational barriers related to the availability of drugs in pharmacies. Recommended drugs (nitrofurantoin) were often not available in the preferred user friendly dosages:

*"I think you may conclude that nitrofurantoin - twice a day 100 mg - is not available in the Netherlands. And as a result we have a problem in practice with user convenience. I think that is a serious problem"*.

### Suggested interventions to improve adherence

Interventions mentioned to address these barriers were to increase the availability of recommended drugs (Table 3). GPs urged that nitrofurantoin should be available in a more user friendly dosage.

"It's simple: get furabid [nitrofurantoin] back in the preferred dosage of twice a day 100 mg!"

In addition, GPs suggested that guidelines should be pilot tested regionally by determining the bacterial resistance pattern of the recommended drugs.

*"National guidelines are okay, but you need to test them locally to find out whether they are applicable"*.

## Discussion

In this focus group study we identified the main barriers to the implementation of a national guideline on uncomplicated UTI perceived by Dutch GPs and explored interventions that could address these barriers. We found that the recommendations on both diagnosis and treatment were difficult to follow in practice and determined a specific set of barriers that needs to be addressed to improve adherence. Although GPs were aware of the recommendations, attitudinal and external barriers prevented them from following the recommendations consistently in practice. The care concerning UTI could be improved, if these barriers are sufficiently addressed. Several interventions for overcoming these barriers were suggested by the GPs, providing opportunities for guideline developers, implementers, and GPs in practice.

With regard to diagnosing uncomplicated UTI, one of the main barriers was that GPs disagreed with the recommendation because they argued the supporting evidence. Previous studies showed that adherence to recommendations based on scientific evidence is higher than to recommendations that are not supported with evidence [30,31]. However, providing evidence-based recommendations in guidelines is not enough. More efforts are needed to raise awareness among GPs with the evidence supporting the recommendations and to convince them with strong arguments why they should change their current practice. Discussing the recommendations in peer review groups may be a useful method as the effectiveness of interactive small group education has been demonstrated [32,33]. Since the barriers are mainly related to attitude, an educational program addressing GPs' attitudes in addition to knowledge transfer, may be particularly effective [34-36].

Organisational constraints to performing dipslides in out of hour services were also mentioned as barriers. Some GPs perceived the use of dipslides in general as inconvenient and do not have a supply in practice. This is consistent with other Dutch studies showing that GPs hardly use the dipslide in case of a negative nitrite test, particularly in out of hours services [6,37]. A suggested intervention is to adapt the recommendation to current practice, i.e. not using dipslides in out of hours services, which is more consistent with guidelines in other countries [4]. Another option, not mentioned in our focus group session, is to hand dipslides over to the patients and ask them to show it the next day in out of hours services (Saturday; Sunday) or to the own GP (Monday). Although the dipslide has high diagnostic accuracy, the guideline could also offer alternative options for diagnosis in specific circumstances. Improving the organisation and coordination in out of hours services by developing local protocols and agreements with hospitals was also suggested by the GPs.

One of the barriers to implementing the treatment recommendation on uncomplicated UTI in practice was a perceived lack of applicability due to local patterns of bacterial resistance. Bacterial resistance to commonly prescribed antibiotics in uncomplicated UTIs has been increasing in recent years [38-40] and resistance patterns have been found to differ significantly between regions [41]. As a result, national guidelines may not always be regionally applicable. Although some regional variation in bacterial resistance in general practices in the Netherlands was reported in 2004 [42], up to date and conclusive evidence for the existence of such variation is not available. However, it seems useful to pilot test guidelines by systematically monitoring the regional resistance patterns. If there is strong variation, the recommendations in the guideline could be regionally adapted to specific patterns of resistance.

Another barrier perceived by GPs is that drug dosages recommended in the guideline are not always available at pharmacies. Some GPs did not want to prescribe drugs that need to be taken four times a day because of user inconvenience, and therefore do not prescribe the drug of first choice. It would be helpful if guideline developers consider the availability of drug dosages to optimize the implementability of recommendations. Negotiation with national pharmacy organisations may be helpful to reach these goals.

By focusing on the individual recommendations within the guideline, we were able to gain an in-depth understanding of the barriers and the interventions needed to address them. The use of a predefined framework of barriers to implementation triggered physicians to think about a broad range of barriers and potential interventions to improve guideline acceptance and guideline adherence [28]. Our approach appeared to be useful in exploring a wide range of barriers and potential interventions and had an educational effect as well [22]. Moreover, by involving the target group of GPs in exploring interventions to address these barriers, we expect that the feasibility and effectiveness of interventions will improve. These methods can be applied in implementation programs on a range of topics and in other settings as well.

A limitation of our study is that we organised only one focus group. The participants were motivated GPs and those with a positive attitude towards guidelines may be overrepresented. However, our sample of GPs does correspond quite well in terms of basic characteristics to the total population of Dutch GPs. In addition, by offering accreditation points to the GPs, creating an incentive to participate for less motivated GPs as well, we attempted to reduce this bias. Secondly, the number of participants in our study was limited, making it difficult to quantify our findings. However, our aim was to explore the relevant barriers qualitatively instead of quantifying their relative importance. As our sample seems to be representative in terms of basic characteristics, we assume having described a substantial variation in barriers and interventions perceived by Dutch GPs. An electronic survey among a larger sample of GPs will follow to quantify our findings.

We only included GPs in our focus group session, while guideline adherence often also depends on other staff members in general practice. Changing habits and routines of practice assistants may be as difficult as those of GPs. Specific protocols and educational sessions for practice assistants may be useful. Quality improvement programs, involving all practice staff, such as NHG Practice Accreditation (NPA), could facilitate this process [43].

Finally, our study was based on a Dutch guideline questioning the generalisability of our findings to other countries. Guidelines on UTI in different countries differ substantially, particularly concerning diagnosis recommendations [4]. For example, most guidelines do not recommend the use of dipslides. Interventions to address barriers regarding this method may therefore not be relevant. However, barriers regarding topics such as the organisation of care in out of hours services will be relevant to other countries as well, as management of UTI often happens out of hours. Problems with antibiotic resistance patterns and availability of drugs also apply to other countries. Regional pilot testing of the guideline may be useful in many countries, even in smaller ones. Moreover, our methods used to determine barriers to implementation among guideline users are applicable in other countries as well.

## Conclusions

Despite GPs' awareness of the guideline recommendations, our study showed that several attitudinal and external barriers prevented them from consistently following the recommendations on uncomplicated UTI in practice. Guideline implementation could be improved if guideline developers and implementers are aware of the potential barriers and involve all relevant staff members in the implementation strategies. Educational programs addressing providers' attitudes in addition to knowledge transference, and improving the coordination and organisation of care, could improve adherence to the guideline on uncomplicated UTI. Involving the target group in selecting useful interventions to implement the guideline recommendations may improve the feasibility and success of implementation strategies.

## Competing interests

The authors declare that they have no competing interests.

## Authors' contributions

ML was involved in designing and conducting the focus group study, in analysing and interpreting the data and in drafting the manuscript. JB was involved in designing and conducting the focus group study and critically revising the manuscript. JZ participated in analysing the data. GW supervised the study, participated in the design of the study and helped to draft the manuscript. All authors read and approved the final manuscript.

## Pre-publication history

The pre-publication history for this paper can be accessed here:

http://www.biomedcentral.com/1471-2296/11/51/prepub
